# Extrapleural pneumonectomy plus rib resection for malignant pleural mesothelioma: a case report

**DOI:** 10.1186/s13019-014-0176-7

**Published:** 2014-11-18

**Authors:** Yoshinori Yamashita, Hiroaki Harada, Hidenori Mukaida, Mayumi Kaneko

**Affiliations:** Director of General Thoracic Surgery, Kure Medical Center and Chugoku Cancer Center, 3-1 Aoyama, Kure, 737-0023 Japan; Department of General Thoracic Surgery, Hiroshima City Asa Hospital, Hiroshima, Japan; Department of Pathology, Hiroshima City Asa Hospital, Hiroshima, Japan

**Keywords:** Malignant pleural mesothelioma, Chest wall invasion, Extrapleural pneumonectomy

## Abstract

We report a case of a 58-year-old Japanese man with epithelioid-type MPM, with a left chest wall protruding tumor, who underwent extrapleural pneumonectomy and chest wall resection. Postsurgical pathology showed that surgical margins were free of mesothelioma cells. Positron emission tomography taken 5 years after surgery showed no recurrence at any site, suggesting that long-term survival could be dependent on margin-free tumor resection or that specific MPM subgroups have lower malignant potential, leading to improved survival.

**Electronic supplementary material:**

The online version of this article (doi:10.1186/s13019-014-0176-7) contains supplementary material, which is available to authorized users.

## Background

Chemotherapy, immunotherapy, irradiation, hyperthermia, and surgery have all been applied for malignant pleural mesothelioma (MPM); however, presently, with the exception of surgery, the outcomes of these treatments are unsatisfactory. The benefit of extrapleural pneumonectomy (EPP) for MPM has been controversial since data from the MARS feasibility trial were published [1]. However, in three phase 2 trials of EPP, as part of trimodality treatment, 50–71% of patients completed the planned treatment [2]. Many chest surgeons consider clinical T1-2 N0 disease to be an indication for EPP, although this is not widely propounded because of the malignant potential of MPM and the surgical stress associated with EPP. Here, we report the case of a patient with stage III MPM who experienced long-term survival after radical EPP with simultaneous chest wall resection.

## Case presentation

**Figure 1:**
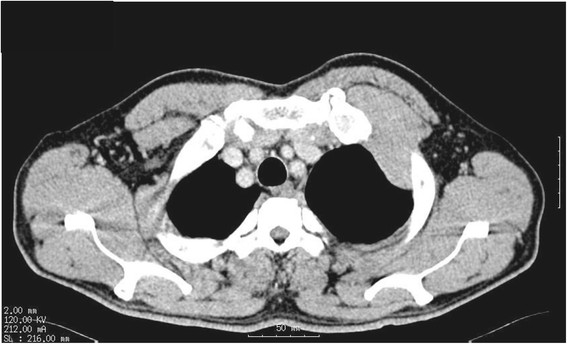
**Preoperative chest computed tomography findings after the drainage of effusion.** A chest wall tumor invading the space between the first and second ribs.

A 58-year-old Japanese man was admitted with left chest pain. He worked in the electrical industry for 40 years. Chest radiography revealed left pleural effusion and a 6-cm diameter protruding tumor on the anterior chest wall. A needle biopsy pathologically confirmed epithelioid-type MPM. Computed tomography revealed direct intercostal invasion of mesothelioma between the first two ribs (Figure 1). Preoperative OK-432 (Picibanil^TM^, Chugai Pharm. Co. Ltd., Tokyo, Japan) was administered twice (total 3KE) intrathoracically to promote adhesion between the visceral and the parietal pleura. We decided to perform EPP on consultation with the patient, following a diagnosis of clinical T3N0M0 stage III MPM based on the International Mesothelioma Interest Group classification.

**Figure 2:**
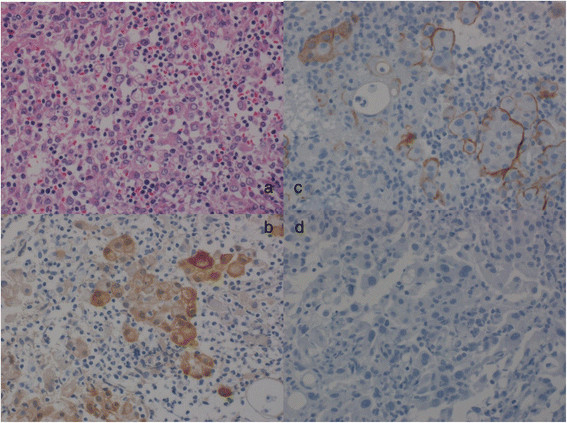
**Histopathological and immunohistochemical examination of the resected tumor revealed epithelioid-type malignant pleural mesothelioma. a**. Hematoxylin and eosin staining at the site of chest invasion shows malignant pleural mesothelioma and inflammatory cells. **b**. Calretinin positivity. **c**. D2-40 positivity. **d**. Thyroid transcription factor-1 negativity.

Two weeks after OK-432 infusion, EPP plus mediastinal lymph node dissection, combined with chest wall resection, including the first two ribs, the pericardium, and the diaphragm, was performed via a sigmoid incision of the fifth intercostal space. The pericardium and the diaphragm were reconstructed using polytetrafluoroethylene Blood loss and the duration of surgery were 1085 g and 490 minutes, respectively. Histological analysis showed that, the mesothelioma cells reached the fascia of the major pectoral muscle invading the intercostal muscles and partially destroying the ribs. However, pathological investigation confirmed that all the surgical margins were tumor-free. In addition, microscopic findings showed the aggregation of inflammatory cells, lymphocytes, plasma cells, and histiocytes with the mesothelioma cells (Figure 2). Postoperative immunohistochemical analysis confirmed the diagnosis of epithelioid-type MPM. Mesothelioma cells stained positive for calretinin, D2-40, CAM5.2, vimentin, and myoglobin, but were negative for PE-10, TTF-1, and CEA. The Ki67 and p53 labeling indices of the mesothelioma cells were 30–40% and 10%, respectively.

**Figure 3:**
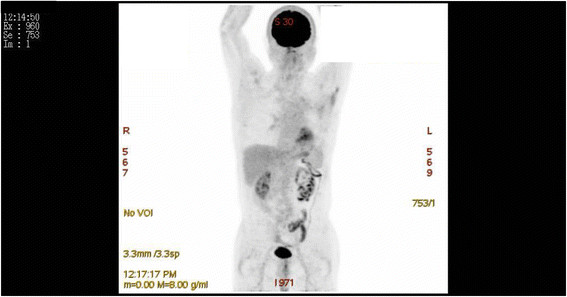
**A positron emission tomography scan shows that the patient remains recurrence free 5 years after extrapleural pneumonectomy.**

The patient refused postoperative adjuvant chemotherapy and radiation therapy, although this was recommended. The patient has resumed his daily routine and has been followed-up for 64 months. The most recent positron emission tomography scan revealed no postsurgical recurrence at any site for >5 years (Figure 3).

## Discussion

The histological and immunohistochemical findings presented here undoubtedly revealed epithelioid-type MPM. Although the reason for the aggregation of inflammatory cells was unknown, this was presumed to be attributable to OK-432 infusion into the thoracic cavity before surgery. To avoid the adverse events and the surgical stress caused by EPP, pleurectomy and decortication (P/D) has been suggested as a more feasible alternative that would confer substantial survival benefit. Lang-Lazdunski et al. [3] reported that P/D combined with hyperthermic pleural lavage with povidone-iodine and adjuvant chemotherapy was well tolerated and associated with low morbidity and mortality. On the other hand, retrospective analysis of the International Association for the Study of Lung Cancer Mesothelioma database showed no difference in the median survival time between EPP and P/D, except in stage I patients, in whom EPP conferred a substantial survival benefit [4].

Some reports indicated that trimodality improves long-term survival [2]. However, there are examples in the literature of long-term survival in the absence of trimodal treatment [5]-[7]. There are 3 possibilities for long term-survival here. First, tumor cell-free clean surgical margins is essential for a good prognosis. Therefore, a successful curative surgery should be based on careful evaluation of the surgical margins. Here, 36 paraffin-embedded blocks of the pathological specimen were examined, all of which revealed surgical margins free from mesothelioma cells, including those taken from the pericardium, diaphragm, and chest wall. Second, the pathological diagnosis of MPM is not consistent and the classification of mesothelioma is heterogeneous. Some subgroups of patients with MPM may have a good prognosis [5]-[7]. It is desirable that an exact diagnosis and classification of mesothelioma should be established to predict long-term prognosis. This essential information would be invaluable for determining the optimal surgical indication and for patient selection, especially for EPP. Third, OK-432 helps to promote adhesion between the visceral pleura and the parietal pleura. This adhesion prevents leakage of pleural effusion in the event that the visceral pleura is accidentally opened during EPP. However, OK-432 promotes the aggregation of lymphocytes, which could exhibit antitumor effects [8].

## Conclusion

The patient here demonstrates long-term, recurrence-free survival following EPP, with combined resection of a chest wall-invading T3 tumor, for MPM. Our case findings suggest that long-term survival could be dependent on margin-free tumor resection and that even in advanced cases, some patients with a subgroup of MPM that has lower malignant potential may have a better prognosis than others.

## Consent

Written informed consent was obtained from the patient for publication of this case report and any accompanying images. A copy of the written consent is available for review by the Editor-in-Chief of this journal.

## Authors’ contributions

YY, HM and HH are the doctors for the general thoracic surgery. YY wrote the article. YY and HM performed the surgery and assisted the post operative care. HH is the consultant of performing the study. MK assessed the pathological findings. All authors read and approved the final manuscript.

## Authors’ original submitted files for images

Below are the links to the authors’ original submitted files for images. Authors’ original file for figure 1 Authors’ original file for figure 2 Authors’ original file for figure 3

## References

[1] Treasure T, Lang-Lazdunsski L, Walke D, Bliss JM, Tan C, Entwisle J, Snee M, O’Brien M, Thoma G, Senan S, O’byme K, Kibum LS, Spicer J, Landau D, Edwards J, Coombes G, Dalison L, Peto J (2011). Extra-pleural pneumonectomy versus mesothelioma clinical outcomes of the Mesothelioma and Radical Surgery (MARS) randomized feasibility study. Lancet Oncol.

[2] Hiddinga BI, van Meerbeeck JP (2013). Surgery in Mesothelioma, where do we go after MARS?. J Thorac Oncol.

[3] Lang-Lazdunski L, Bille A, Belcher E, Cane P, Landau D, Steele J, Taylor H, Spicer J (2011). Pleurectomy/decortication, hyperthermic pleural lavage with povidone-iodine followed by adjuvant chemotherapy in patients with malignant pleural mesothelioma. J Thorac Oncol.

[4] Rusch VW, Giroux D, Kennedy C, Ruffini E, Cangir AK, Rice D, Pass H, Asamura H, Waller D, Edwards J, Weder W, Hoffmann H, van Meerbeeck JP (2012). Initial analysis of the international association for the study of lung cancer mesothelioma database. J Thorac Oncol.

[5] Nomori H, Horio H, Kobayashi R, Morinaga S (1997). Long survival after extrapleural pneumonectomy for pleural malignant mesothelioma with metastasis to infradiaphragmatic lymph node. Scand Cardiovasc J.

[6] Bitchatchi E, Kayser K, Perelman M, Richter ED (2010). Mesothelioma and asbestosis in a young woman following occupational asbestos exposure: Short latency and long survival: case report. Diagn Pathol.

[7] Okonogi N, Ebara T, Ishikawa H, Yoshida D, Ueno M, Maeno T, Suga T, Nakano T (2012). A seven-year disease-free survivor of malignant pleural mesothelioma treated with hyperthermia and chemotherapy: a case report. J Med Case Rep.

[8] Lafreniere R, Borkenhagen K, Bryant LD (1990). In vivo administration of picibanil (OK-432) prior to tumor harvest leads to an enhancement of tumor-infiltrating lymphocyte (TIL) cytotoxicity. J Surg Oncol.

