# Supplementary figures and images for: Extrapleural pneumonectomy plus rib resection for malignant pleural mesothelioma: a case report
Source: J Cardiothorac Surg. 2014 Nov 18;9:176. doi: 10.1186/s13019-014-0176-7 (PMC4241212; doi:10.1186/s13019-014-0176-7)

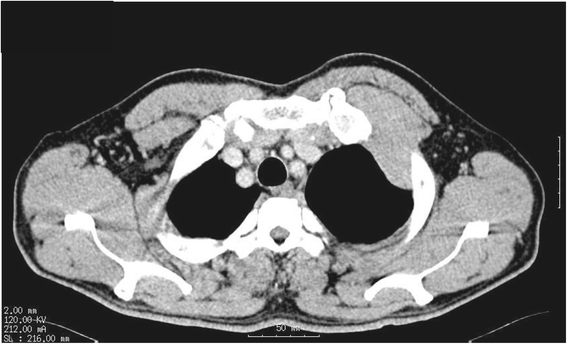

Supplement: Supplementary file 1 — Authors’ original file for figure 1 [file 13019_2014_176_MOESM1_ESM.gif]

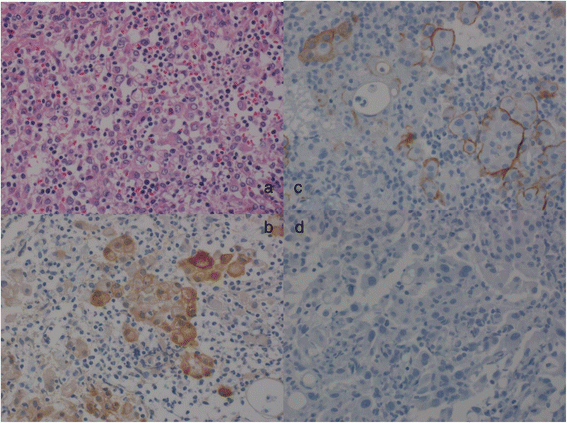

Supplement: Supplementary file 2 — Authors’ original file for figure 2 [file 13019_2014_176_MOESM2_ESM.gif]

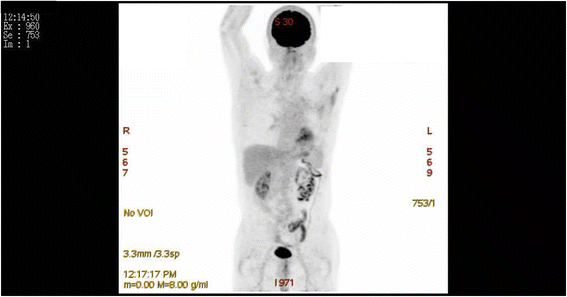

Supplement: Supplementary file 3 — Authors’ original file for figure 3 [file 13019_2014_176_MOESM3_ESM.gif]
